# Thanatophoric Dysplasia Type I Confirmed by Fibroblast Growth Factor Receptor 3 (FGFR3) Mutation: Clinical Course and Ethical Considerations

**DOI:** 10.7759/cureus.106363

**Published:** 2026-04-03

**Authors:** Maria I Soares, Patrícia Veríssimo, Catarina Coelho, Raquel A Branco, Mónica Marçal

**Affiliations:** 1 Pediatrics Department, Hospital José Joaquim Fernandes, Unidade Local de Saúde do Baixo Alentejo, Beja, PRT; 2 Pediatrics Department, Hospital de Santarém, Unidade Local de Saúde da Lezíria, Santarém, PRT; 3 Pediatrics Department, Hospital de Santo André, Unidade Local de Saúde da Região de Leiria, Leiria, PRT; 4 Pediatrics Department, Unidade Local de Saúde da Arrábida, Hospital de São Bernardo, Setúbal, PRT; 5 Pediatrics Department, Neonatal Intensive Care Unit, Unidade Local de Saúde de Lisboa Ocidental, Hospital de São Francisco Xavier, Lisboa, PRT

**Keywords:** fgfr3 mutation, genetic counseling, neonatal outcome, palliative care, prenatal diagnosis, skeletal dysplasia, thanatophoric dysplasia, type i td

## Abstract

Thanatophoric dysplasia (TD) is the most common lethal skeletal dysplasia, caused by de novo fibroblast growth factor receptor 3 (FGFR3) mutations. Prenatal ultrasound may detect key features such as severe micromelia, narrow thorax, macrocephaly, and temporal lobe dysplasia, although molecular confirmation is essential. Type I TD (TD1), the most frequent subtype, shows “telephone-receiver” femur bowing, frontal bossing, and midface hypoplasia. Type II TD presents with a cloverleaf skull and straight femurs. TD is generally fatal due to pulmonary hypoplasia, narrow thorax, and brainstem compression, with survival beyond the early neonatal period being uncommon. We report a newborn with prenatal suspicion of skeletal dysplasia, confirmed postnatally as TD1 via FGFR3 p.Ter807Trp mutation, highlighting the importance of prenatal counseling, early genetic confirmation, and palliative care involvement.

## Introduction

Thanatophoric dysplasia (TD) is the most common lethal skeletal dysplasia, with an estimated prevalence of 0.24-0.69 per 10,000 births. It results from a de novo autosomal-dominant mutation in the fibroblast growth factor receptor 3 (FGFR3) gene. Typical features include severe micromelia, narrow thorax, macrocephaly, thick and redundant skin folds, platyspondyly, and temporal lobe dysplasia [1]. TD comprises two subtypes: type I (80%) and type II (20%) [2]. Type I TD (TD1) has an estimated incidence of one in 60,000 births [3,4]. It is characterized by "telephone-receiver" femoral bowing, frontal bossing, and midface hypoplasia [5-7].

The accuracy of prenatal diagnosis based on ultrasonography is variable, ranging from 40% to 88% [2,8]. Second-trimester ultrasound generally allows early suspicion of TD, particularly by identifying long-bone shortening and deep, abnormal transverse sulci in the temporal lobes. Polyhydramnios occurs in approximately 50% of cases [9]. Definitive diagnosis requires molecular testing. Several skeletal dysplasias share overlapping prenatal features, including FGFR3*-*related conditions such as achondroplasia and severe achondroplasia with developmental delay and acanthosis nigricans, as well as genetically distinct entities such as achondrogenesis and campomelic dysplasia. These conditions carry markedly different prognoses and recurrence risks. Therefore, precise genotypic characterization is essential for clinical management and genetic counseling [2,5,8,10,11].

TD is frequently associated with late fetal or early neonatal death, primarily due to respiratory failure secondary to pulmonary hypoplasia and a narrow thorax. Compression of the brainstem due to narrowing of the foramen magnum is another described mechanism [3,12,13]. Survival beyond the first 48 hours without organ support is rare, although isolated cases have been reported, creating significant challenges in prenatal counseling [14].

This case aims to contribute to the limited literature on the natural history of TD1, while highlighting the clinical, ethical, and communication challenges that arise when molecular confirmation is not established prenatally and when cultural and linguistic barriers intersect, shaping the overall management of the patient and family.

## Case presentation

**Figure 1:**
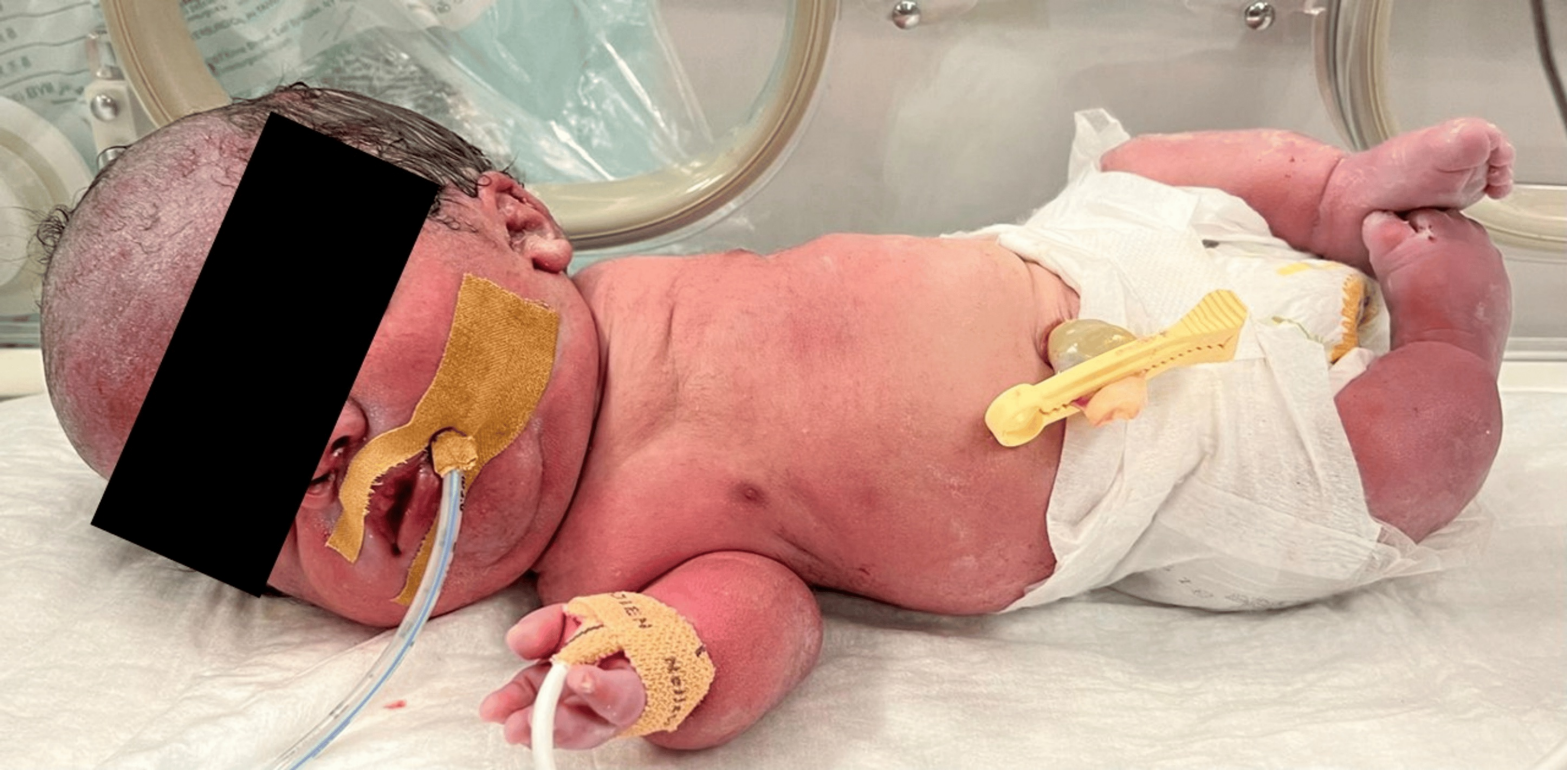
Clinical features of type I thanatophoric dysplasia Neonate demonstrating macrocephaly, frontal bossing, depressed nasal bridge, short neck, narrow thorax, micromelia, and bowed legs

We report the case of a Pakistani woman in her 30s, gravida 2, para 1 (child alive and healthy), with a history of hypothyroidism controlled with levothyroxine, in a nonconsanguineous marriage. The pregnancy was monitored, and serologies were unremarkable. Ultrasound at 24 weeks of gestation revealed long bones below the third percentile, curved femurs and humeri, a narrow thorax, pronounced lumbar lordosis, and a head circumference at the 95th percentile. These findings, suggestive of skeletal dysplasia, raised suspicion of achondroplasia, prompting referral to a fetal pathology consultation. The parents declined molecular testing and chose to continue the pregnancy. Fetal echocardiography at 32 weeks showed mild ventricular disproportion with right-sided predominance and asymmetry between the pulmonary artery and the aorta, without major structural heart disease. Ultrasound at 35 weeks continued to show findings consistent with skeletal dysplasia, including a marked spinal hyperextension, abnormal facial features, and low-set ears; amniotic fluid volume and Doppler studies remained normal. An emergency cesarean section was performed at 35 weeks and four days due to suspected fetal distress and was complicated by an intraoperative uterine rupture. Apgar scores were 1, 6, and 8 at 1, 5, and 10 minutes, respectively. Endotracheal intubation was performed, and positive-pressure ventilation with a maximum FiO₂ of 100% was initiated. The newborn exhibited initial gasping at three minutes and regular respiratory movements by 10 minutes, and was admitted to the neonatal intensive care unit (NICU). According to the Fenton 2013 growth charts [15], birth weight was 2,160 g (10th-50th percentile), body length 36 cm (less than third percentile), and head circumference 36.5 cm (>97th percentile), with characteristic features of TD1 (Figure 1).

Echocardiography confirmed disproportion of the great vessels. Serial transfontanelle cranial ultrasounds revealed widening of the interhemispheric fissure and subarachnoid space; diffuse, bilateral, and symmetrical increased echogenicity of the white matter; and prominent lateral ventricles.

Genetic testing revealed the FGFR3 variant NM_000142.5:c.2421A>G p.(*807Trpext*101), classically known as p.Term807Trp, which underlies TD1 [16]. This variant was classified as pathogenic according to American College of Medical Genetics and Genomics/Association for Molecular Pathology guidelines criteria PS4, PM2, PM4, PP4, and PP5 [17], consistent with its classification in ClinVar-Variation ID: 65564 [18].

Taken together, the key distinguishing features in this case were consistent with a diagnosis of TD1: severe rhizomelic micromelia with characteristic "telephone-receiver" femur bowing, macrocephaly with frontal bossing and midface hypoplasia, a markedly narrow thorax, and molecular confirmation of a pathogenic FGFR3 variant. The absence of a cloverleaf skull and the presence of bowed, rather than straight, femurs allowed clinical differentiation from TD type II. The combination of clinical, radiological, and genetic findings, evaluated in a multidisciplinary setting, was essential for establishing a definitive diagnosis and guiding subsequent management.

The patient remained in the NICU dependent on respiratory support. Care planning was supported by multiple multidisciplinary discussions involving the neonatology and palliative care teams and the parents throughout the hospitalization. These meetings focused on prognosis, goals of care, and the proportionality of life-sustaining interventions. As the clinical course evolved and ventilatory weaning proved unachievable, these discussions ultimately guided a shared transition toward comfort-directed care, with a focus on the avoidance of artificial life-prolonging measures and on providing continuous support to both the newborn and the family. Despite supportive management, death occurred on day 30 of life following an episode of refractory hypoxemia and profound bradycardia.

## Discussion

The term "thanatophoric dysplasia" is derived from Greek and means "death-bearing" [4]. TD is widely regarded as a lethal condition, primarily due to respiratory failure caused by pulmonary hypoplasia and a narrow thorax, or due to bulbar compression resulting from foramen magnum stenosis [11]. Cases of prolonged survival are uncommon and generally associated with TD1 presenting with an atypical phenotype, raising important ethical dilemmas [14,19]. The few cases described have provided valuable insights into phenotypic progression and prognosis, although most individuals remain dependent on invasive ventilation and have significant neurodevelopmental impairment [19].

Survival beyond the neonatal period in TD is exceedingly rare but not impossible. Carroll et al. reported a long-term survivor with TD1 who, despite requiring invasive mechanical ventilation, achieved meaningful developmental milestones, including social interaction and communication, challenging the unconditional classification of TD as uniformly lethal [14]. Nikkel et al. similarly described a 25-year follow-up of a TD survivor who, while profoundly dependent on ventilatory support and with significant neurodevelopmental impairment, maintained a degree of cognitive engagement [19]. These cases illustrate that survival is contingent upon the provision of aggressive, long-term technological support, and underscore the importance of individualized counseling that neither overstates nor minimizes the burden of such interventions. In the present case, the inability to achieve ventilatory weaning was consistent with the natural history of the disease and precluded consideration of long-term life-sustaining measures.

TD1 must be differentiated from other lethal skeletal dysplasias that present with micromelia and a narrow thorax, including severe achondroplasia, achondrogenesis, and campomelic dysplasia [20]. Osteogenesis imperfecta type II and the short rib-polydactyly syndromes may also mimic the presentation, but can be distinguished by the presence of multiple fractures or polydactyly, respectively. Other diagnoses to consider include atelosteogenesis, hypochondrogenesis, and perinatal hypophosphatasia [11]. Radiologically, TD1 is characterized by bowed femurs, platyspondyly, and a small, cone-shaped thorax, in contrast to type II TD, which presents with a cloverleaf skull and straight femurs. Definitive diagnosis is established by identifying heterozygous pathogenic variants in FGFR3 [5].

Fetal ultrasound can detect limb shortening as early as 13 weeks of gestation. Multiple studies have demonstrated that this examination allows prenatal suspicion of TD in 50%-88% of cases. Confirmation relies on molecular testing, either through chorionic villus sampling, amniocentesis, or, more recently, analysis of cell-free fetal DNA in maternal blood [2]. Establishing a definitive diagnosis is essential for accurate genetic counseling, enabling informed parental decision-making regarding pregnancy management and future reproductive risk. In TD, recurrence risk is similar to that of the general population, given its origin as a de novo mutation [3,20]. Postnatally, clinical and radiological features are sufficiently characteristic and often allow a high degree of diagnostic certainty [20].

Prenatal counseling in cases of suspected lethal skeletal dysplasia presents unique and multifaceted challenges. The rarity of the condition, the complexity of the phenotypic spectrum, and the inherent uncertainty prior to molecular confirmation make it difficult for clinicians to provide precise prognostic information at the time of initial diagnosis [1]. Parents are often confronted with deeply consequential decisions, including the option of termination of pregnancy, expectant management, or preparation for palliative care, in a context of significant diagnostic ambiguity and emotional distress. Guidelines recommend that counseling be nondirective, multidisciplinary, and culturally sensitive, incorporating input from maternal-fetal medicine, clinical genetics, neonatology, and palliative care teams [1].

In the present case, the parents were informed during the second trimester that the fetus had a skeletal dysplasia. However, the full severity spectrum of the condition may not have been adequately understood at that stage, as the diagnosis had not yet been molecularly confirmed, and the phenotypic spectrum of skeletal dysplasias is broad. The language barrier may have been one of several factors contributing to this limited understanding, though the extent to which it influenced their decision to decline molecular testing remains uncertain. Diagnostic confirmation by genetic analysis occurred 30 days after birth, during which time the neonatology and palliative care teams provided continuous support in preparing the parents for the expected prognosis of TD, given the strong clinical suspicion.

The delay in molecular confirmation, while not altering the clinical management in this case, raised important ethical considerations. During the interval between clinical suspicion and genetic confirmation, the medical team faced decisions regarding the intensity of neonatal intervention in the absence of a definitive diagnosis. This reflects a broader ethical tension in neonatal medicine: the obligation to provide life-sustaining treatment until a diagnosis is confirmed, balanced against the potential for prolonging suffering in conditions with a near-certain lethal prognosis. Transparent and iterative communication with the family during this period, acknowledging uncertainty while conveying clinical conviction, is essential to maintaining trust and facilitating shared decision-making.

The management of these patients should include ongoing involvement of the palliative care team, which plays a fundamental role in supporting families and helping them understand the expected prognosis, although this can be particularly challenging in conditions as rare as TD. Families must be informed about late manifestations of TD when discussing potential treatment options. In this case, the inability to achieve ventilatory weaning reinforced the guarded prognosis and the inherent lethality of the condition in the absence of invasive life-sustaining interventions.

## Conclusions

TD type I is a rare but severe skeletal disorder caused by de novo mutations in the FGFR3 gene. Respiratory failure due to pulmonary hypoplasia is the leading cause of early neonatal mortality. Prenatal ultrasonography can detect key phenotypic features, including severe limb shortening, macrocephaly, frontal bossing, midface hypoplasia, a narrow thorax, and the characteristic “telephone-receiver” femur bowing, which allow early recognition and timely referral for specialized assessment. Definitive diagnosis requires molecular confirmation of pathogenic FGFR3 variants, which is essential for accurate genetic counseling and for guiding families regarding prognosis and management options. Clear and direct communication about expected outcomes, treatment limitations, and potential life-sustaining interventions is a fundamental aspect of care, ensuring that the family is well informed and supported throughout the process. A coordinated, multidisciplinary approach involving neonatology, clinical genetics, and palliative care is indispensable to provide both clinical guidance and compassionate support to the affected infant.
